# Effect of Speleotherapy on Dyspnea, Quality of Life, and Functional Status in Patients With Chronic Airway Diseases

**DOI:** 10.7759/cureus.107441

**Published:** 2026-04-21

**Authors:** Zehra Karaaslan, Ertan Kucuksayan, Aslinur Sircan-Kucuksayan

**Affiliations:** 1 Health Tourism, Institute of Graduate Education, Alanya Alaaddin Keykubat University, Antalya, TUR; 2 Medical Biochemistry, Faculty of Medicine, Alanya Alaaddin Keykubat University, Antalya, TUR; 3 Biophysics, Faculty of Medicine, Alanya Alaaddin Keykubat University, Antalya, TUR

**Keywords:** asthma, chronic obstructive pulmonary disease, complementary therapy, dyspnea, speleotherapy

## Abstract

Background

Speleotherapy is a complementary non-pharmacological intervention based on exposure to the stable microclimatic conditions of natural caves. However, clinical evidence evaluating its effects on patient-reported outcomes remains limited. This study aimed to evaluate the effects of speleotherapy on dyspnea severity, quality of life, and functional status in patients with chronic airway diseases.

Methods

A total of 28 voluntary participants aged 18-65 years with a confirmed diagnosis of asthma or chronic obstructive pulmonary disease (COPD) were evaluated in this prospective pre-post observational study. Clinical outcomes were assessed using the modified Medical Research Council (mMRC) Dyspnea Scale, St. George’s Respiratory Questionnaire (SGRQ), Short-Form 36 Health Survey (SF-36), and Beck Anxiety Inventory (BAI) before and after the speleotherapy sessions. Data were analyzed using IBM SPSS Statistics 25.0 (IBM Corp., Armonk, NY). Paired t-test or Wilcoxon signed-rank test was applied where appropriate, and p < 0.05 was considered statistically significant.

Results

After speleotherapy, patients demonstrated statistically significant improvements in all evaluated domains. mMRC dyspnea scores decreased from 3.32 ± 1.31 to 1.89 ± 0.96 (p < 0.01). Total SGRQ scores improved from 63.27 ± 25.51 to 14.19 ± 9.50 (p < 0.01). SF-36 subscales revealed increased physical, emotional, and social functioning (p < 0.01 for all). Anxiety levels also significantly decreased according to the Beck scale (p < 0.05).

Conclusion

Speleotherapy was associated with clinically meaningful improvements in dyspnea severity, health-related quality of life, and functional status among patients with asthma and COPD. These findings suggest that cave-based microclimate therapy may serve as a supportive non-pharmacological intervention within multidisciplinary management strategies for chronic respiratory diseases.

## Introduction

Chronic airway diseases, primarily asthma and chronic obstructive pulmonary disease (COPD), represent a major cause of morbidity and mortality worldwide. Asthma affects more than 262 million people worldwide and causes substantial mortality, while COPD remains one of the leading causes of death globally [1,2]. In Turkey, the prevalence of COPD has increased over recent decades, driven by population aging, tobacco exposure, air pollution, and occupational risk factors [3,4]. Similarly, asthma remains one of the most common chronic respiratory conditions in Turkey, with prevalence estimates ranging between 7% and 9%, depending on demographic characteristics [5,6]. Asthma and COPD are characterized by persistent respiratory symptoms, including chronic cough, sputum production, and progressive dyspnea [7,8]. These physical manifestations lead to significant airflow limitations, which directly impair exercise capacity and restrict the performance of daily activities [9]. Asthma and COPD, although distinct clinical entities, may share overlapping features such as persistent airflow limitation, airway inflammation, and similar symptom profiles, a condition often referred to as asthma-COPD overlap (ACO), which may complicate clinical interpretation and management. Beyond these physiological constraints, patients frequently experience a profound psychological burden, most notably elevated levels of anxiety and distress, often stemming from the chronic fear of breathlessness [10,11]. The synergistic effect of functional impairment and psychological distress results in a comprehensive deterioration of health-related quality of life (QoL), affecting physical, emotional, and social functioning [12].

Despite advances in pharmacological therapies and guideline-based management, a considerable proportion of patients with chronic airway diseases continue to seek complementary and non-pharmacological interventions to alleviate persistent symptoms and improve QoL. Speleotherapy, also referred to as subterranean climate therapy, is one such approach that involves controlled exposure to the stable microclimatic conditions of natural caves for therapeutic purposes [13]. This modality is based on the use of naturally occurring environmental conditions rather than active drug administration and is generally classified as an environment-based complementary therapy. While some components of this microclimate can be partially replicated in artificial halotherapy settings, the complex and stable conditions of natural caves cannot be fully reproduced. While some components of this microclimate can be partially replicated in artificial halotherapy settings, the complex and stable conditions of natural caves cannot be fully reproduced.

The therapeutic effects of speleotherapy are characterized by a specific microclimate that is associated with stable temperature, low concentrations of pollutants and allergens, high relative humidity, and a specific ratio of elements such as Na+, Cl-, Mg+2, and Ca+2 ions [14]. Several studies suggest that cave microclimates may exert beneficial effects on respiratory function in asthma and COPD patients [15-17]. High relative humidity may reduce airway irritation and facilitate mucus clearance, which may contribute to symptom relief. The absence of common aeroallergens and particulate matter can provide hypoallergenic and immunoprotective effects [18]. Additionally, some caves may have mildly elevated CO₂ levels, which could contribute to symptom relief by reducing airway resistance in the short term; studies have shown that CO₂ inhalation or experimentally induced hypercapnia can acutely relax airways and decrease respiratory resistance [19,20]. These mechanisms provide a theoretical basis for the potential clinical effects of speleotherapy.

The present study was conducted in Damlataş Cave (Antalya, Turkey), an environment characterized by stable temperature, high humidity, and low allergen load, conditions considered relevant for respiratory rehabilitation. Although speleotherapy has been practiced for decades, clinical evidence supporting its efficacy remains scarce. In particular, data evaluating both respiratory and psychosocial outcomes using validated clinical instruments are limited. Therefore, this study aimed to evaluate the clinical effects of speleotherapy on dyspnea, QoL, and anxiety in patients with asthma and COPD. Validated assessment tools were used, including the modified Medical Research Council (mMRC) Dyspnea Scale, St. George’s Respiratory Questionnaire (SGRQ), the Short-Form 36 Health Survey (SF-36), and the Beck Anxiety Inventory (BAI).

## Materials and methods

Study design and participants

This prospective observational study was conducted to evaluate the effects of speleotherapy on symptom severity, dyspnea, and QoL in patients with chronic airway diseases. The study protocol was approved by the Alanya Alaaddin Keykubat University Clinical Research Ethics Committee. The study population consisted of volunteers who visited Damlataş Cave for therapeutic purposes and had a confirmed clinical diagnosis of asthma or COPD. A total of 28 participants who met the inclusion criteria were enrolled. All participants provided written informed consent before their inclusion in the study. Participants were included if they were between 18 and 65 years of age, had a confirmed diagnosis of asthma or COPD, and voluntarily agreed to participate in the study. Participants were excluded if they had an acute respiratory infection, severe cardiovascular disease, or pregnancy. Participants were not regular attendees of speleotherapy; most were undergoing the therapy for the first time or had only occasional exposure in the distant past.

Speleotherapy protocol

Speleotherapy was conducted in Damlataş Cave (Alanya, Turkey), a natural cave traditionally used for supportive respiratory therapy. Participants attended daily speleotherapy sessions lasting approximately two to four hours per day for a duration of 10-20 consecutive days. During each session, participants remained seated on benches or performed light activities. Participants were instructed to maintain their usual medical therapy throughout the intervention.

Data collection instruments

Clinical outcomes were assessed immediately before the initiation of speleotherapy and immediately after the completion of the treatment program. All questionnaires were administered in their validated Turkish versions by face-to-face interview. All clinical scales were administered once before the initiation of the speleotherapy program and once after the completion of the full treatment period; no daily repeated measurements were performed. Demographic data were assessed using a 23-item form that evaluated age, gender, education, and medical history. 

mMRC Dyspnea Scale

The severity of dyspnea was assessed using the Medical Research Council (MRC) dyspnea scale, a simple and widely used five-grade scale originally derived from the MRC classification of breathlessness described by Fletcher et al. [20]. The MRC scale grades dyspnea based on the level of physical activity that provokes breathlessness, ranging from 0 (no dyspnea except with strenuous exercise) to 4 (severe dyspnea that limits basic daily activities). The MRC dyspnea scale has been extensively used in clinical and epidemiological studies of chronic airway diseases [21].

St. George’s Respiratory Questionnaire

Health-related QoL was assessed using the SGRQ, a disease-specific instrument developed by Jones et al. to evaluate health status in patients with chronic airway diseases, including asthma and COPD [22]. The questionnaire consists of 50 items grouped into three domains: symptoms (eight items), activity (16 items), and impacts (26 items). The SGRQ evaluates respiratory symptoms, activity limitation due to breathlessness, and the overall impact of the disease on daily life. Scores for each domain and the total score range from 0 to 100, with higher scores indicating worse health-related QoL. The Turkish version of the SGRQ has been shown to be valid and reliable, with its psychometric properties established by Polatlı et al. in 2013 [23].

Short-Form 36 Health Survey

The SF-36 was developed within the Medical Outcomes Study to assess health-related QoL across a wide range of clinical and population settings. The questionnaire consists of 36 items that measure eight health domains: physical functioning, role limitations due to physical health (Role Physical), bodily pain, general health perceptions, vitality (energy/fatigue), social functioning, role limitations due to emotional problems (Role Emotional), and mental health (psychological well-being and distress). Higher scores indicate better perceived health status [24].

Beck Anxiety Inventory

The BAI is a self-report measure developed by Beck and colleagues in 1988 to assess the severity of anxiety symptoms in individuals [25]. The inventory consists of 21 items rated on a four-point Likert scale ranging from 0 to 3, with higher scores indicating greater levels of anxiety. The Turkish version of the BAI was shown to be valid and reliable in a study conducted by Ulusoy et al. in 1998 [26].

All assessment tools used in this study (MRC Dyspnea Scale, SGRQ, SF-36, and BAI) are established instruments widely used in clinical research and were applied with appropriate citation of the sources.

Statistical analysis

Data analysis was performed using IBM SPSS Statistics Software (IBM Corp., Armonk, NY). The normality of distribution was evaluated using Kolmogorov-Smirnov and Shapiro-Wilk tests. Descriptive statistics were presented as mean ± standard deviation or frequency (n, %). For pre- and post-speleotherapy comparisons, the paired sample t-test was used for normally distributed data, and the Wilcoxon signed-rank test was utilized for non-normal distributions. A p-value of <0.05 was considered statistically significant.

## Results

A total of 28 participants were included in the study. The mean age of the study population was 48.7 ± 13.8 years, and 20 participants (71.4%) were female. Regarding diagnosis, 21 patients (75.0%) had asthma, and seven patients (25.0%) had COPD. The duration of respiratory disease was one to five years in six patients (21.4%), six to 10 years in six patients (21.4%), 11-20 years in nine patients (32.1%), and more than 21 years in seven patients (25.0%). With respect to smoking status, 12 participants (42.8%) were never-smokers, nine (32.1%) were former smokers, and seven (25.0%) were current smokers. Baseline demographic and clinical characteristics of the participants are summarized in Table 1.

**Table 1:** Baseline demographic and clinical characteristics of the study participants.

Variable	Total (n = 28)
Age (years), mean ± SD	48.7 ± 13.8
Sex, n (%)	
Female	20 (71.4%)
Male	8 (28.6%)
Diagnosis, n (%)	
Asthma	21 (75.0%)
COPD	7 (25.0%)
Disease duration (years), n (%)	
1-5 years	6 (21.4%)
6-10 years	6 (21.4%)
11-20 years	9 (32.1%)
> 21 years	7 (25.0%)
Smoking status, n (%)	
Never	12 (42.8%)
Former	9 (32.1%)
Current	7 (25.0%)

Dyspnea severity

**Figure 1:**
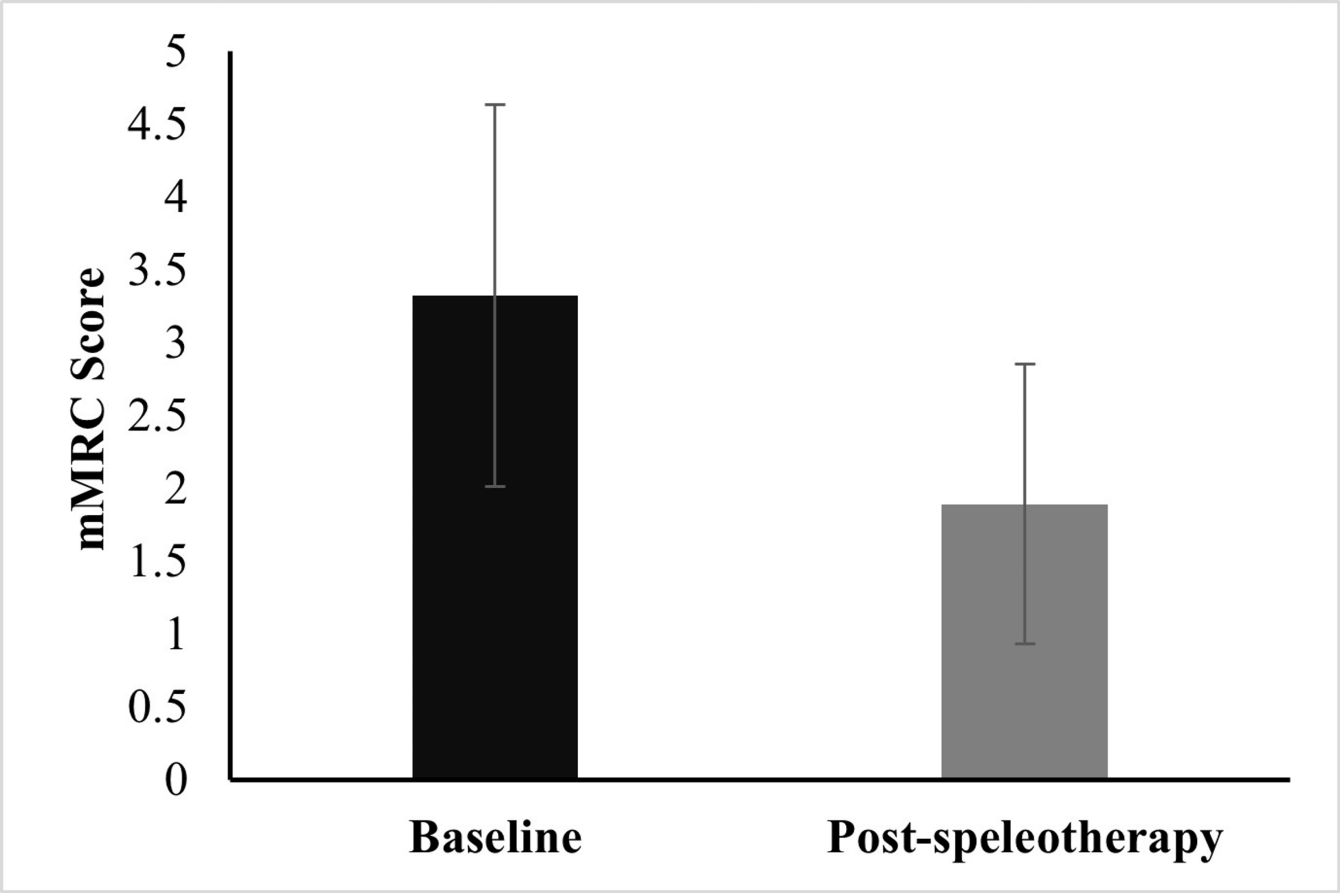
Comparison of mMRC Dyspnea Scale scores before and after speleotherapy. Statistical analysis was performed using the Wilcoxon signed-rank test due to non-normal data distribution (p < 0.01). mMRC: modified Medical Research Council Dyspnea Scale [20]

Dyspnea severity, assessed using the mMRC Dyspnea Scale, showed a significant change following the speleotherapy intervention. Mean mMRC scores decreased from 3.32 ± 1.31 at baseline to 1.89 ± 0.96 after the intervention (<0.01). These findings indicate an improvement in patient-reported dyspnea after speleotherapy (Figure 1).

Disease-specific quality of life

Disease-specific QoL was assessed using the SGRQ. Significant improvements were observed in all SGRQ domains following speleotherapy (Table 2). In the symptom domain, the mean score decreased from 63.39 ± 25.01 before speleotherapy to 27.38 ± 11.13 after the intervention, indicating a statistically significant improvement (p < 0.001). Similarly, the activity limitation domain demonstrated a marked reduction in mean scores, decreasing from 72.65 ± 24.28 at baseline to 20.33± 17.80 after speleotherapy (p < 0.001). In the impact domain, mean scores declined significantly from 57.88 ± 28.95 before speleotherapy to 6.56 ± 5.81 following the intervention, reflecting a substantial reduction in the perceived effects of the disease on daily life (p < 0.001).

**Table 2:** Changes in SGRQ scores before and after speleotherapy. SGRQ: St. George’s Respiratory Questionnaire [22]

SGRQ domain	Baseline	Post-speleotherapy	Test	Z-value	p-value
Symptom	63.39 ± 25.01	27.38 ± 11.13	Wilcoxon signed-rank test	Z = -2.82	< 0.01
Activity	72.65 ± 24.28	20.33 ± 17.80	Wilcoxon signed-rank test	Z = -3.54	< 0.01
Impact	57.88 ± 28.95	6.56 ± 5.81	Wilcoxon signed-rank test	Z = -4.78	< 0.01

**Figure 2:**
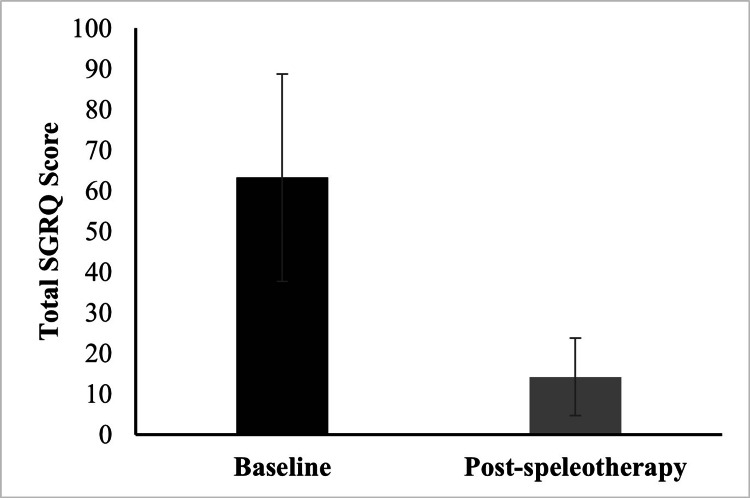
Total SGRQ scores before and after speleotherapy. Statistical analysis was performed using the Wilcoxon signed-rank test due to non-normal data distribution (p < 0.01). SGRQ: St. George’s Respiratory Questionnaire [22]

Consistent with these findings, the total SGRQ score showed a significant decrease from 63.27 ± 25.51 at baseline to 14.19 ± 9.50 after speleotherapy (p < 0.001), indicating an overall improvement in disease-specific QoL (Figure 2).

General quality of life

Assessment of general health-related QoL using the SF-36 questionnaire revealed improvements in several domains following speleotherapy (Table 3). The physical functioning domain showed a statistically significant increase, with mean scores rising from 42.14 ± 26.30 before speleotherapy to 76.80 ± 18.57 after the intervention (p < 0.001). The Role Physical domain showed a statistically significant increase, with mean scores rising from 28.68 ± 31.75 before speleotherapy to 93.75 ± 21.11 after the intervention (p < 0.001). The Role Emotional domain, mean scores increased markedly from 33.30 ± 30.80 at baseline to 80.30 ± 19.67 after speleotherapy, indicating a significant improvement (p < 0.001). The vitality (energy/fatigue) domain demonstrated a significant increase in mean scores, from 42.86 ± 19.83 before speleotherapy to 66.96 ± 12.35 after the intervention (p < 0.001). Similarly, the mental health domain showed a statistically significant improvement, with mean scores increasing from 60.00 ± 15.81 to 75.29 ± 11.52 following speleotherapy (p < 0.001).

In the social functioning domain, mean scores rose from 43.14 ± 27.95 before the intervention to 76.46 ± 15.87 after speleotherapy, reflecting a significant enhancement in social functioning (p < 0.001). The bodily pain domain also demonstrated a significant increase, with mean scores improving from 61.39 ± 25.83 at baseline to 85.11 ± 15.78 after speleotherapy (p < 0.001). Finally, the general health perception domain showed a marked improvement, with mean scores increasing from 31.07 ± 23.47 before speleotherapy to 65.71 ± 16.54 after the intervention (p < 0.001).

**Table 3:** Changes in SF-36 Health Survey domain scores before and after speleotherapy. SF-36: Short-Form 36 Health Survey [24]

SF-36 domain	Baseline	Post-speleotherapy	Test	Z-value	p-value
Physical functioning	42.14 ± 26.30	76.80 ± 18.57	Wilcoxon signed-rank test	Z = 2.64	< 0.01
Role physical	28.68 ± 31.75	93.75 ± 21.11	Wilcoxon signed-rank test	Z = 3.94	< 0.01
Role emotional	33.30 ± 30.80	80.30 ± 19.67	Wilcoxon signed-rank test	Z = 3.22	< 0.01
Vitality	42.86 ± 19.83	66.96 ± 12.35	Wilcoxon signed-rank test	Z = 2.13	< 0.01
Mental health	60.00 ± 15.81	75.29 ± 11.52	Wilcoxon signed-rank test	Z = 1.99	< 0.01
Social functioning	43.14 ± 27.95	74.46 ± 15.87	Wilcoxon signed-rank test	Z = 2.51	< 0.01
Bodily pain	61.39 ± 25.83	85.11 ± 15.78	Wilcoxon signed-rank test	Z = 2.01	< 0.01
General health	31.07 ± 23.47	65.71 ± 16.54	Wilcoxon signed-rank test	Z = 2.93	< 0.01

Anxiety levels

**Figure 3:**
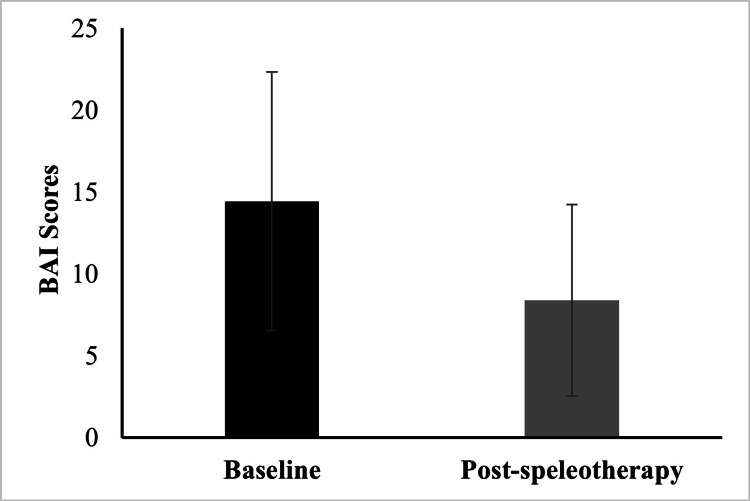
BAI scores before and after speleotherapy. Statistical analysis was performed using the Wilcoxon signed-rank test due to non-normal data distribution (p < 0.01). BAI: Beck Anxiety Inventory [25]

Psychological assessment using the BAI demonstrated a significant reduction in anxiety scores following speleotherapy. Mean BAI scores decreased from 14.43 ± 7.91 at baseline to 8.38 ± 5.86 post-intervention (p < 0.01), indicating an improvement in anxiety symptoms (Figure 3).

## Discussion

Speleotherapy is a complementary approach that utilizes the stable microclimatic characteristics of natural caves and underground salt mines and has been used as a supportive therapeutic option, particularly in chronic airway diseases such as asthma and COPD. The theoretical basis of this intervention relies on the potential physiological effects of constant temperature, high relative humidity, low aeroallergen and particulate load, mineral ion content, and mildly elevated CO₂ levels observed in some cave environments. High relative humidity may enhance airway surface liquid hydration and support mucociliary clearance, while reduced particulate exposure may decrease epithelial irritation. Additionally, stabilized environmental conditions may limit bronchial hyperreactivity. Mild elevations in CO₂ have been shown under experimental conditions to exert acute bronchodilatory effects and temporarily reduce airway resistance. Together, these mechanisms provide a plausible physiological basis for symptomatic relief associated with speleotherapy. However, it should be emphasized that most mechanistic explanations remain based on experimental or indirect clinical observations rather than direct physiological measurements obtained during cave exposure.

However, a substantial proportion of speleotherapy and halotherapy studies reported in the literature are methodologically heterogeneous. Many investigations are single-center studies lacking randomization, blinding, or appropriate control groups. A comprehensive review conducted in the context of COPD emphasized the limited number of rigorously designed trials and highlighted the need for well-conducted randomized controlled studies [27]. Therefore, current evidence does not establish definitive efficacy but rather suggests a moderate-to-low level of evidence indicating a potential clinically meaningful signal.

Pilot studies conducted in COPD populations have reported reductions in symptom burden, improvements in anxiety levels, and enhanced exercise tolerance following speleotherapy. In the study by Mętel et al., performed in older adults undergoing pulmonary rehabilitation in an underground salt mine environment, significant improvements were observed in functional fitness parameters, including endurance and dynamic balance, while lung function indices were not the primary outcome measures of the study [28]. Likewise, studies conducted in asthma populations have demonstrated improvements in symptom control and quality-of-life measures following speleotherapy or related subterranean interventions, despite variable effects on objective inflammatory markers such as fractional exhaled nitric oxide (FeNO) and spirometric parameters [14]. These findings suggest that the therapeutic impact of speleotherapy may be more consistently reflected in patient-reported outcomes and functional performance measures rather than in airflow-based physiological parameters alone. Our findings are consistent with this body of literature. We observed a marked reduction in mMRC dyspnea scores and statistically significant improvements in both total and subscale SGRQ scores, indicating better disease-specific QoL following the intervention. However, the observed effect size exceeds the average changes reported in previous studies, which may partly reflect short-term post-intervention assessment and the absence of a control group. Therefore, although clinically encouraging, our findings should be interpreted with appropriate caution.

In asthma research, randomized clinical trials that incorporated speleotherapy-based interventions have demonstrated time-dependent effects on inflammatory biomarkers such as FeNO, although clear and consistent group-level superiority has not been demonstrated across all outcome measures [14]. Improvements in asthma control and quality-of-life parameters, however, have been reported. These findings indicate that while the effects of speleotherapy-related interventions on inflammatory biomarkers may be heterogeneous, improvements may be more consistently observed in patient-reported symptom control and functional capacity measures. In our study, the significant increase in the physical functioning domain of the SF-36 aligns with this pattern of predominantly patient-centered benefit.

Recent investigations have also explored speleotherapy in emerging clinical contexts such as post-COVID syndrome. In a recent study, no significant improvements in diffusing capacity of the lung for carbon monoxide (DLCO) or symptom burden were observed following speleotherapy, highlighting the uncertainty of its physiological effects in post-COVID syndrome [29]. However, evidence in this area is still limited, and proposed mechanisms require further experimental confirmation.

The observed improvements in dyspnea and QoL in our cohort may be explained by multiple interacting mechanisms: (i) reduced exposure to aeroallergens and particulate matter, (ii) facilitation of secretion clearance through high humidity, (iii) interruption of the anxiety-dyspnea cycle frequently observed in chronic airway diseases, and (iv) psychophysiological relaxation associated with time spent in a controlled and isolated environment. It is also possible that daily attendance and increased health awareness during the intervention period contributed to perceived improvements. The concurrent improvements in anxiety and exercise tolerance reported in COPD studies support this multidimensional effect model. However, it should be emphasized that speleotherapy has primarily been investigated as a complementary intervention alongside standard medical treatment rather than as a direct alternative to pharmacological therapy.

Several limitations of the present study must be acknowledged. The absence of a control group prevents exclusion of placebo effects. Additionally, the inclusion of both asthma and COPD patients introduces clinical heterogeneity, as these conditions may share overlapping features while remaining distinct disease entities. The lack of long-term follow-up limits conclusions regarding the sustainability of observed benefits. Objective inflammatory biomarkers were not assessed, and therefore, the mechanistic interpretation remains indirect. Consequently, causal inferences cannot be established. Future research should include adequately powered randomized controlled designs with extended follow-up periods and simultaneous assessment of spirometric parameters, inflammatory markers, and patient-reported outcomes. Subgroup analyses comparing asthma and COPD populations separately would also strengthen disease-specific interpretation.

## Conclusions

In conclusion, our findings suggest that speleotherapy may serve as a supportive adjunct within a multidisciplinary management framework to improve symptom control and QoL in patients with chronic airway diseases. In clinical practice, it may be considered as a rehabilitative component in stable-phase patients with appropriate selection criteria. Nonetheless, stronger evidence from rigorously designed trials is required before definitive clinical recommendations can be made. 
